# DNA Methyltransferase 1 (DNMT1) Function Is Implicated in the Age-Related Loss of Cortical Interneurons

**DOI:** 10.3389/fcell.2020.00639

**Published:** 2020-07-22

**Authors:** Anne Hahn, Daniel Pensold, Cathrin Bayer, Jessica Tittelmeier, Lourdes González-Bermúdez, Lisa Marx-Blümel, Jenice Linde, Jonas Groß, Gabriela Salinas-Riester, Thomas Lingner, Julia von Maltzahn, Marc Spehr, Tomas Pieler, Anja Urbach, Geraldine Zimmer-Bensch

**Affiliations:** ^1^Department of Functional Epigenetics, Institute of Human Genetics, University Hospital Jena, Jena, Germany; ^2^Department of Functional Epigenetics in the Animal Model, Institute of Biology II, RWTH Aachen University, Aachen, Germany; ^3^Research Training Group 2416 MultiSenses – MultiScales, RWTH Aachen University, Aachen, Germany; ^4^Transcriptome and Genome Analysis Laboratory (TAL), Department of Developmental Biochemistry, University of Göttingen, Göttingen, Germany; ^5^Leibniz Institute on Aging – Fritz Lipmann Institute (FLI), Jena, Germany; ^6^Department of Chemosensation, Institute of Biology II, RWTH Aachen University, Aachen, Germany; ^7^Centre for Nanoscale Microscopy and Molecular Physiology of the Brain (CNMPB), Department of Developmental Biochemistry, University of Göttingen, Göttingen, Germany; ^8^Institute of Neurology, University Hospital Jena, Jena, Germany

**Keywords:** aging, inhibitory interneurons, GABA, cerebral cortex, synapse, proteostasis, DNA methylation, transcriptional control

## Abstract

Increased life expectancy in modern society comes at the cost of age-associated disabilities and diseases. Aged brains not only show reduced excitability and plasticity, but also a decline in inhibition. Age-associated defects in inhibitory circuits likely contribute to cognitive decline and age-related disorders. Molecular mechanisms that exert epigenetic control of gene expression contribute to age-associated neuronal impairments. Both DNA methylation, mediated by DNA methyltransferases (DNMTs), and histone modifications maintain neuronal function throughout lifespan. Here we provide evidence that DNMT1 function is implicated in the age-related loss of cortical inhibitory interneurons. *Dnmt1* deletion in parvalbumin-positive interneurons attenuates their age-related decline in the cerebral cortex. Moreover, conditional *Dnmt1*-deficient mice show improved somatomotor performance and reduced aging-associated transcriptional changes. A decline in the proteostasis network, responsible for the proper degradation and removal of defective proteins, is implicated in age- and disease-related neurodegeneration. Our data suggest that DNMT1 acts indirectly on interneuron survival in aged mice by modulating the proteostasis network during life-time.

## Introduction

Aging mediates structural, neurochemical, and physiological alterations in the brain that are associated with behavioral changes, memory decline, and cognitive impairments (Rozycka and Liguz-Lecznar, 2017). Cognitive aging results in metabolic, hormonal and immunological dysregulation, increased oxidative stress and inflammation, altered neurotransmission and synaptic plasticity as well as reduced neurotrophic support of neurons (Rozycka and Liguz-Lecznar, 2017). Notably, in the aging brain, distinct cell types and circuits are affected differently (reviewed in Zimmer-Bensch, 2019a).

Inhibitory interneurons of the cerebral cortex are key players in cortical information processing (Kann et al., 2014) and particularly affected by aging. Reduced interneuron numbers were reported across diverse species and cortical regions (reviewed in Zimmer-Bensch, 2019a). Additionally, morphological abnormalities and dysfunction of GABAergic synapses emerge as major factors in aging-related impairments of nervous system function (Morrison and Baxter, 2012). These findings confirm previous reports of declined inhibition (Shetty and Turner, 1998; Stanley and Shetty, 2004; Cheng and Lin, 2013). In line with reduced neurotransmitter release, major changes in the expression of genes related to neurotransmission and transcriptional repression of GABA-related transcripts have been described for the human prefrontal cortex (Loerch et al., 2008), but also in brains across different mammalian species (reviewed in Zimmer-Bensch, 2019a). Diminished expression of genes involved in synaptic function indeed appears to be a conserved feature of mammalian brain aging (Jiang et al., 2001; Loerch et al., 2008; Ianov et al., 2016).

Given the importance of GABAergic inhibitory interneurons in cortical information processing, age-associated defects in inhibitory circuits contribute to cognitive decline and age-related disorders (Rozycka and Liguz-Lecznar, 2017). Such defects include the loss of synaptic contacts, decreased GABA release, and reduced postsynaptic responsiveness, thus disturbing the excitation/inhibition balance in the aging brain. Fast-spiking parvalbumin (PV) positive interneurons represent the most abundant subset of cortical inhibitory interneurons (Druga, 2009). They execute both feedforward and feedback inhibition, and are responsible for generating gamma-frequency oscillations (Sohal et al., 2009; Buzsáki and Wang, 2012; Kann et al., 2014; Willems et al., 2018). In schizophrenia patients, a reduction in PV interneurons and their dysfunction have been associated with the loss of gamma oscillations, manifesting in working memory and executive function deficits (Torrey et al., 2005; Sohal et al., 2009). Upon aging, PV interneurons are diminished in cell numbers in the somatosensory, auditory, and motor cortices of rats as well as in the hippocampus (Miettinen et al., 1993; Ouda et al., 2008). Moreover, altered PV interneuron function is implicated in age-related diseases like Alzheimer’s disease (AD; Rossignol, 2011; Verret et al., 2012). Together, these studies emphasize the role of PV interneurons in cortical function. Hence, detailed analysis of age-related changes in this interneuron subpopulation might help to understand the processes underlying cognitive aging and age-related memory impairments.

Apart from synaptic defects, aging is accompanied by a declining proteostasis network that causes ineffective protein degradation, which can lead to neuronal death (Douglas and Dillin, 2010). Lysosomal degradation is critical for removing defective proteins or protein aggregates delivered by autophagy- or endocytosis-triggered endosomal pathways (Nixon and Cataldo, 1995; Nixon et al., 2000; Winckler et al., 2018). Moreover, lysosomal dysfunction is associated with age-related neurodegenerative pathologies like Parkinson’s and Alzheimer’s disease (Zhang et al., 2009; Carmona-Gutierrez et al., 2016). Another protein removal pathway is built upon inclusion into multivesicular bodies (MVBs) and exosome release (Riva et al., 2019). The latter has recently been implicated in contributing to neurodegenerative disease and mental disorders (Bellingham et al., 2012; Delpech et al., 2019; Saeedi et al., 2019).

At the molecular level, epigenetic mechanisms emerge as crucial players in the physiology of healthy aging and the pathophysiology of age-related neurological disorders. Epigenetic mechanisms involve inheritable as well as reversible chromatin modifications, including DNA methylation and histone modifications, which influence gene transcription and post-transcriptional events (Fuks, 2005). Further epigenetic key players are represented by non-coding RNAs, which can act on transcriptional, post-transcriptional, and translational level (Geisler and Coller, 2013; Cech and Steitz, 2014; Zimmer-Bensch, 2019b).

DNA methylation executed by DNA methyltransferases (DNMTs) affects gene expression through diverse mechanisms (Maunakea et al., 2010; Gelfman et al., 2013; Lyko, 2018) and is implicated in the pathogenesis of brain aging (Cui and Xu, 2018). We have recently found that DNMT1-dependent DNA methylation modulates synaptic function of cortical PV interneurons by acting on endocytosis-mediated vesicle recycling (Pensold et al., 2020). Since regulation of both synaptic function and DNA methylation are involved in brain aging, we here investigate whether DNMT1-dependent transcriptional control in PV interneurons contributes to their age-related defects.

## Materials and Methods

### Animals

The following mouse strains were used: C57BL/6 wild-type mice and transgenic mice on the C57BL/6 background including *Pvalb-Cre*/*tdTomato*/*Dnmt1* control as well as *Pvalb-Cre*/*tdTomato*/*Dnmt1 loxP*^2^ mice. The transgenic mice were established by crossing the *Pvalb-Cre* line (obtained from Christian Huebner, University Hospital Jena, Germany and described in Hippenmeyer et al., 2005) with the *tdTomato* transgenic reporter mice (obtained from Christian Huebner, University Hospital Jena, Germany and described in Madisen et al., 2010) and the *Dnmt1 loxP^2^* mice (B6;129Sv-Dnmt1^TM 4*Jae*^/J, Jaenisch laboratory, Whitehead Institute; United States). The *Dnmt1 loxP^2^* mice have LoxP-sites flanking exons 4 and 5 of the *Dnmt1* gene. To avoid germline recombination due to instable Cre expression in sperm, as already described for this *Pvalb-Cre* line (Kobayashi and Hensch, 2013), only maternal Cre inheritance was permitted. For this, males from the *tdTomato* line or *tdTomato*/*Dnmt1 loxP^2^* line were cross-bred with Cre-positive females of the *Pvalb-Cre*/*tdTomato* or *Pvalb-Cre*/*tdTomato*/*Dnmt1 loxP*^2^ lines to achieve the *Pvalb-Cre*/*tdTomato*/*Dnmt1* control and *Pvalb-Cre*/*tdTomato*/*Dnmt1 loxP*^2^ mice, respectively. Cre-positive males were used for experiments but not for further breeding. Transgenic *Pvalb-Cre*/*tdTomato*/*Dnmt1* control and *Pvalb-Cre*/*tdTomato*/*Dnmt1 loxP*^2^ mice are abbreviated as *Dnmt1* WT (*wild-type*) and *Dnmt1* KO (*knockout*) in the figures, respectively. Both lines were parallel back-crossed over more than 8 generations. CRE-mediated deletion leads to out-of-frame splicing from exon 3 to exon 6, resulting in a null *Dnmt1* allele (Jackson-Grusby et al., 2001). The floxed *Dnmt1* allele was genotyped with forward GGGCCAGTTGTGTGACTTGG and reverse CCTGGGCCTGGATCTTGGGGA primer pairs resulting in a 334 bp WT and 368 bp mutant band. The *tdTomato* allele was genotyped using the following set of four primers: WT forward AAGGGAGCTGCAGTGGAGTA, WT reverse CCGA AAATCTGTGGGAAGTC, mutant forward CTGTTCCTGTAC GGCATGG, mutant reverse CTGTTCCTGTACGGCATGG giving WT (297 bp) and mutant (196 bp) bands. The *Pvalb*-*Cre* genotyping was performed by applying AAACGTT GATGCCGGTGAACGTGC forward and TAACATTCTCCC ACCGTCAGTACG reverse primer resulting in a 214 bp fragment. All animal procedures were performed in strict compliance with the EU directives 86/609/EWG and 2007/526/EG guidelines for animal experiments and were approved by the local government (Thüringer Landesamt, Bad Langensalza, Germany). Animals were housed under 12 h light/dark conditions with *ad libitum* access to food and water.

### Ladder Rung Test

Cohorts of *Pvalb-Cre/tdTomato*/*Dnmt1* control as well as *Pvalb-Cre/tdTomato*/*Dnmt1 loxP*^2^ mice were consecutively tested over different ages starting from 3 to 21 months. Mice were placed onto a ladder beam (transparent) with rungs in a regular pattern (every 10 mm) at a slight incline (∼30°) with the home box at the end. Time to cross the ladder was measured, not including the time spent in a stop or walking back toward the starting point. The scoring system according to Metz and Whishaw (2009) was used for foot placement accuracy. In each test session the animals had to cross the ladder consecutively for three times.

### Isolation and Primary Cultivation of Dissociated Embryonic Single Cells

Pregnant dams were anesthetized by an intraperitoneal injection of 50% chloral hydrate in phosphate buffered saline (PBS; pH 7.4; 2.5 μg chloral hydrate per g body weight). After death of the dam, all embryos were dissected out of both uterine horns and instantly decapitated. The brain was dissected in ice-cold and sterile filtered Gey’s Balanced Salt Solution (GBSS; 1.53 mM CaCl_2_, 3.66 mM KCl, 0.22 mM KH_2_PO_4_, 1.03 mM MgCl_2_^∗^6H_2_O, 0.28 mM MgSO_4_^∗^7H_2_O, 137.93 mM NaCl, 2.702 mM NaHCO_3_, 0.84 mM Na_2_HPO_4_, and 5.56 mM D(+)-Glucose).

Dissociated embryonic medial ganglionic eminence (MGE)-derived single cells for primary culture were prepared from MGE explants dissected from coronal brain sections according to Zimmer et al. (2011). Briefly, embryonic brains were prepared in Krebs buffer (126 mM NaCl, 2.5 mM KCl, 1.2 mM NaH_2_PO_4_, 1.2 mM MgCl_2_, 2.1 mM CaCl_2_, 10 mM D(+)-Glucose, and 12.5 mM NaHCO_3_), embedded in 4% low-melt agarose (Carl Roth, Germany) at 37°C for coronal sectioning with a vibratome at 4°C. MGE explants were collected in ice-cold Hank’s Balanced Salt Solution (HBSS; Invitrogen, United States) supplemented with 0.65% D(+)-Glucose. After incubation with 0.04% trypsin (Invitrogen) in HBSS for 17 min at 37°C, cells were dissociated by trituration and filtering through nylon gauze (pore size 140 μm; Millipore).

Dissociated neurons were plated on coverslips coated with 19 μg/mL laminin (Sigma-Aldrich, Germany) and 5 μg/mL poly-L-lysine (Sigma-Aldrich) at a density of 225 cells/mm^2^ in Neurobasal Medium (Thermo Fisher Scientific) supplemented with 1xB27 (Thermo Fisher Scientific), 100 U/mL penicillin, 100 μg/mL streptomycin, and 0.5 mM GlutaMax (Thermo Fisher Scientific). After incubation at 37°C, 5% CO_2_ in a humid atmosphere with 95% relative humidity for 7 days *in vitro* (DIV), cells were fixed in 4% paraformaldehyde (PFA) in PBS (pH 7.4) for 10 min at room temperature (RT).

### Cell Culture

Cerebellar granule (CB) cells were cultured in Dulbecco’s Modified Eagle’s Medium with high glucose (DMEM; Invitrogen) supplemented with 10% fetal bovine serum (FBS; Biowest), 1% GlutaMAX, 24 mM of KCl, 100 U/mL penicillin, 100 μg/mL streptomycin incubated at 33°C, 95% relative humidity, 5% CO_2_.

### Transfection With siRNA Oligos and CD63-pEGFP

For siRNA transfections of dissociated embryonic MGE cells of C57BL/6 WT mice and CB cells, reverse lipofection with Lipofectamin© 2000 (Thermo Fisher Scientific, United States) was applied according to the manufacturer’s protocol and as described in Zimmer et al. (2011) using 15 nM control siRNA (BLOCK-iT Alexa Fluor red or green fluorescent oligo, Invitrogen, United States) and 30 nM *Dnmt1* siRNA, *Rab7* siRNA (Santa Cruz Biotechnology) for 5 h in Opti-MEM I Reduced Serum Medium without antibiotics (Thermo Fisher Scientific). MGE-derived neurons were transfected after six DIV, whereas CB cells were plated on coverslips 1 day prior to transfection. Cells were cultured overnight at 37 or 33°C, 5% CO_2_ and 95% relative humidity using the aforementioned cell line specific culture medium prior to fixation.

Transfection for the CD63 overexpression construct was done as described above for siRNA transfection using 2 μg/mL of CD63-pEGFP (Addgene, United States) added for 5 h in Opti-MEM I Reduced Serum Medium (Thermo Fisher Scientific). Cells were cultured overnight at 33°C, 95% relative humidity and 5% CO_2_ using the aforementioned cell line specific culture medium applied to live cell imaging in a petri dish inserted in a chamber heated to 33°C using imaging media of HBSS (Thermo Fisher Scientific) supplemented with 0.65% D(+)-Glucose, 10% FBS, 1% GlutaMAX (Thermo Fisher Scientific), 100 U/mL penicillin, 100 μg/mL streptomycin, and 25 μM HEPES (Thermo Fisher Scientific).

### EGF Endocytosis

Epidermal growth factor (EGF) coupled to Alexa-488 (Molecular Probes, Invitrogen, United States) was used as an endocytic probe. siRNA-transfected CB cells were incubated in serum-free DMEM supplemented with 1% BSA for 1 h at 33°C followed by incubation in uptake media (DMEM, 1% BSA, 50 mM HEPES) containing 0.5 μg/mL EGF coupled to Alexa-488 on ice for 1 h. Cells were then washed 3× with ice-cold PBS (pH 7.4) to remove unbound ligands and then incubated for the indicated time points in serum-free DMEM, 1% BSA 1 h at 33°C. Cells were then put on ice, washed 3× with ice-cold PBS (pH 7.4), then placed in an acid wash [0.2 M acetic acid, 0.5 M NaCl (pH 2.8)] to remove any non-internalized ligands. After fixation in 4% PFA in PBS (pH 7.4) for 10 min, cells were stained against LAMP1.

### Brain Tissue Preparation

Mice were deeply anesthetized by intraperitoneal injection of 50% chloral hydrate in PBS (pH 7.4; 2.5 μg chloral hydrate per g body weight). For *in situ* hybridization experiments, freshly prepared brains were immediately frozen in liquid nitrogen and stored at −80°C. For immunohistochemistry, mice were perfused with PBS (pH 7.4) followed by 4% PFA in PBS (pH 7.4) and brains were dissected. Post-fixation occurred over night at 4°C. Cryoprotection with 10 and 30% sucrose in PBS overnight was applied before freezing in liquid nitrogen and storage at −80°C.

### *In situ* Hybridization, Immunohistochemistry and Immunocytochemistry

For *in situ* hybridizations, adult brains were cryo-sectioned coronally at −20°C (20 μm). *In situ* hybridizations were performed as described by Zimmer et al. (2011) using digoxigenin-labeled riboprobes. The following primers were used to generate the riboprobe: forward GAGAGCTCTGTCGATGACAGACGTGCTC and reverse GA GGTACCTTCTTCAACCCCAATCTTGC for *Pvalb* (NM_01 3645.3). The riboprobe was obtained by *in vitro* transcription using DIG-11-UTP (Roche, Germany) from cDNA fragments cloned in pBluescript II SK (Stratagene, United States). For Nissl staining, adult brains were cryo-sectioned at −20°C (20 μm) and fixed on slides for 30 min in fixation solution [95% (v/v) ethanol and 5% (v/v) acetic acid]. After washing in water, sections were incubated in 0.5% (w/v) cresyl violet for 25 min, and washed in water. Then an ethanol-series (50, 70, and 99%) was applied for 2.5 min each. Subsequently, sections were incubated in xylol for 5 min and mounted in Depex mounting media (Serva, Germany).

For immunocytochemistry on dissociated MGE cells, permeabilization and washing between different incubation steps was performed with 0.1% (v/v) Triton X-100 in PBS (pH 7.4) for 10 min. Blocking with 5% (v/v) normal goat serum in PBS (pH 7.4) was performed for 30 min and primary antibodies were applied overnight at 4°C, secondary antibodies were applied for 1 h. Cells were washed prior to nuclei staining with DAPI (Molecular Probes, United States) for 5 min. CB cells were permeabilized with 0.2% (v/v) Triton X-100 in PBS (pH 7.4) for 10 min prior to blocking with 5% (v/v) normal goat serum in PBS (pH 7.4) for 1 h. Primary antibodies were applied overnight at 4°C, secondary antibodies for 1 h at RT. After nuclei staining with DAPI (Molecular Probes, United States) for 5 min, coverslips were embedded in Mowiol (Carl Roth, Germany). Unless noted differently, all steps were performed at RT.

The following primary antibodies were used: mouse anti-RFP (1:500, Thermo Fisher Scientific), mouse anti-Parvalbumin (1:2,000, Swant Switzerland), rabbit anti-CD63 (1:500, gift from Markus Damme, Biochemisches Institut Christian-Albrechts-Universitaet Kiel), rat anti-LAMP1 (1:200, Thermo Fisher Scientific).

The following secondary antibodies were applied: goat Alexa-488 anti-mouse (1:1,000, Vector), goat Alexa-488 anti-rat (1:1,000, Thermo Fisher Scientific), goat Cy3 anti-mouse (1:1,000, Jackson Immunoresearch), goat Cy5 anti-mouse (1:1,000, Thermo Fisher Scientific), and goat Cy5 anti-rabbit (1:1,000, Thermo Fisher Scientific).

### Isolation of Adult and Aged Cortical Interneurons for FACS

The optimized protocol used to collect the material for DNA and RNA-sequencing was modified based on different protocols (Brewer, 1997; Eide and McMurray, 2005; Brewer and Torricelli, 2007; Saxena et al., 2012). Adult and aged brains were dissected in GBSS (1.53 mM CaCl_2_, 3.66 mM KCl, 0.22 mM KH_2_PO_4_, 1.03 mM MgCl_2_*6H_2_O, 0.28 mM MgSO_4_*7H_2_O, 137.93 mM NaCl, 2.7 mM NaHCO_3_, 0.84 mM Na_2_HPO_4_, 5.56 mM D(+)-Glucose, pH 7.4). Cortical hemispheres were dissected and subsequently handled separately. All following volumes are calculated per cortical hemisphere, which were cut into small pieces and transferred to 5 mL HBSS w/o Ca^2+^ and Mg^2+^ supplemented with 7 mM HEPES, 100 U/mL penicillin, 100 μg/mL streptomycin and 0.65% D(+)-Glucose and washed twice. The tissue was then transferred to 5 mL pre-warmed (20 min at 37°C) Trypsin/EDTA (Life Technologies, United States) supplemented with 132 mM trehalose (Sigma-Aldrich, Germany), 100 U/mL penicillin, 100 μg/mL streptomycin, 10 mM HEPES, and 600 U DNase (Applichem, Germany) and incubated for 30 min at 37°C, rotating the samples every 5 min. Samples were washed with 2.1 mL pre-warmed DMEM/F12 supplemented with 10% FBS, 100 U/mL penicillin, 100 μg/mL streptomycin, and 132 mM trehalose. After adding 0.9 mL pre-warmed HBSS containing 10 mg/mL Collagenase Type 2 (Worthington, United Kingdom) samples were incubated for 25 min at 37°C rotating every 5 min and then washed with 2 mL pre-warmed DMEM/F12 supplemented with 10% FBS, 100 U/mL penicillin, 100 μg/mL streptomycin, 3.3 mM EDTA, and 132 mM trehalose prior to cool down on ice for 2 min. Dissolving of samples occurred in 1.5 mL DMEM/F12 supplemented with 10% FBS, 100 U/mL penicillin, 100 μg/mL streptomycin, and 132 mM trehalose. Trituration was performed using fire-polished and heat-treated (180°C for 8 h) glass capillaries of three different diameters (about 500, 250 μm, and 100 μm), which were coated with DMEM/F12 supplemented with 10% FBS, 100 U/mL penicillin, and 100 μg/mL streptomycin prior to use. Mechanical dissociation was performed by pipetting up and down gently 3–5 times for each diameter starting with the largest, avoiding air bubbles. After each step, the supernatant was collected in 1 mL DMEM/F12 supplemented with 10% FBS, 100 U/mL penicillin, 100 μg/mL streptomycin, and 132 mM trehalose was added to the original sample. After trituration with the smallest glass capillary, the suspension was filtered through nylon gauze (80–100 μm) and centrifuged for 5 min at 160 g, 4°C. After supernatant removal, the pellet was dissolved in 4 mL HBSS w/o Ca^2+^ and Mg^2+^ supplemented with 7 mM HEPES, 100 U/mL penicillin, 100 μg/mL streptomycin, 0.65% D(+)-Glucose and 132 mM trehalose. After centrifugation (5 min, 160 g, 4°C), the pellet was dissolved in PBS (pH 7.4) with 30% Percoll (Sigma-Aldrich, United States) and 132 mM trehalose to perform a density gradient centrifugation for 10 min at 500 g and 4°C. The supernatant was removed and the pellet was dissolved in 250 μL HBSS w/o Ca^2+^ and Mg^2+^ supplemented with 7 mM HEPES, 100 U/mL penicillin, 100 μg/mL streptomycin, 0.65% D(+)-Glucose, and 132 mM trehalose for fluorescence activated cell sorting (FACS).

### FACS Enrichment of tdTomato Cells

Cell suspensions subjected to FACS were prepared from the cortical hemispheres of adult 6 and 18 months old *Pvalb-Cre/tdTomato*/*Dnmt1* control as well as *Pvalb-Cre/tdTomato*/*Dnmt1 loxP*^2^ mice. Following addition of DAPI, cells were sorted using an ARIA III FACS sorter (BD Biosciences, United States) with a maximal flow rate of six. The tdTomato reporter was excited by a 561 nm yellow/green solid-state laser and emission signal was detected in a range of 579 to 593 nm. According to their forward scatter/side scatter criteria (FSC/SSC) followed by cell doublet exclusion via an FSC-H vs FSC-W criterium, DAPI-negative living cells were sorted based on a distinctive tdTomato signal. Cells of interest were collected in HBSS w/o Ca^2+^ and Mg^2+^ supplemented with 7 mM HEPES, 100 U/mL penicillin, 100 μg/mL streptomycin, 0.65% D(+)-Glucose, and 132 mM trehalose at 4°C and pelleted by centrifugation. Enriched tdTomato cells of one hemisphere were prepared for RNA-sequencing, while cells of the contralateral hemisphere were subjected to DNA-isolation for MeDIP-sequencing for each brain used. For RNA isolation, pellets were dissolved in 500 μL Trizol^®^ Reagent (Life Technologies, United States) and subsequently frozen on dry ice. For MeDIP-Seq analysis, cell pellets were frozen at −80°C until further use. Only male mice were used for RNA and MeDIP sequencing.

### RNA/DNA Isolation of Tissue and FAC-Sorted Cells

Adult cortical hemispheres were dissected from whole brain and frozen in liquid nitrogen as described above. For RNA-sequencing, samples were subjected to standard RNA isolation procedure using Trizol^®^ Reagent (Life Technologies, United States). The FACS-enriched tdTomato cells were processed accordingly, with additional application of GlycoBlue (Thermo Fisher Scientific, United States) to a final concentration of 0.2% during RNA precipitation for better visualization of the pellet.

DNA isolation of FACS-enriched tdTomato cells was performed using QIAamp DNA Micro Kit (Qiagen, Germany) according to manufacturer’s instruction and checked for integrity by capillary gel electrophoresis (Bioanalyzer, Agilent Technologies, Inc., United States).

### RNA Sequencing of Adult Cortical Tissue

To reveal potentially relevant genes for age related processes in the brain, we performed RNA sequencing of 6 and 16 months old cortical hemispheres of C57BL/6 mice. The TruSeq RNA Sample Preparation Kit (Illumina, Cat. N°RS-122-2002, United States) was used for library preparation (1 μg total RNA), the QuantiFluor^TM^ dsDNA System (Promega, United States) for quantitation and the DNA 1000 chip on the Bioanalyzer 2100 (Agilent Technologies) to determine the size range of final cDNA libraries prior to amplification and sequencing (cBot and HiSeq2000 from Illumina; PE; 2 × 100 bp; ca. 30 million reads per sample). Sequences were trimmed for adaptor sequences and phred scores <30 via fastq-mcf (ea-utils v1.1.2-484). This data was uploaded to the Galaxy web platform; 2.11.40.6, and we used the public server at *usegalaxy.eu* for further analysis (Afgan et al., 2018). If not stated differently, default settings were applied. Quality check was done via fastqc; v. 0.11.8 (Andrews, 2010) before alignment to the UCSC mouse reference genome mm10 was performed using STAR; v2.7.2b (Dobin et al., 2013) with 2-pass mapping. Reads were aligned to the reference genome using gapped alignment as RNA transcripts are subject to splicing and reads might therefore span distant exons. Data was converted and sorted by samtools; v1.9 (Li et al., 2009). Counting the reads to each gene was done via HTSeq; v0.9.1 (Anders et al., 2015) to the Ensembl gene annotation. Data analysis was performed using R/Bioconductor 3.0.2/2.12 (Luo and Brouwer, 2013); loading DESeq2; v1.22.1 (Love et al., 2014).

Sequence data will be deposited in NCBI’s Gene Expression Omnibus and are accessible through GEO Series upon acceptance of the manuscript.

### RNA Sequencing of FACS-Enriched tdTomato Cells

RNA was isolated using the Trizol^®^ Reagent protocol according to manufacturer’s instructions. RNA quality was assessed by measuring the RIN (RNA Integrity Number) using the fragment analyzer from Advanced Analytical (United States). Library preparation for RNA-Seq was performed using the TruSeq^TM^ RNA Sample Prep Kit v2 (Illumina, Cat. N°RS-122-2002, United States) starting from 50 ng of total RNA. Accurate quantitation of cDNA libraries was performed by using the QuantiFluor^TM^ dsDNA System (Promega, United States). The size range of final cDNA libraries was determined applying the DNA chip on the fragment analyzer (average 350 bp; Advanced Analytical). cDNA libraries were amplified and sequenced by using the cBot and HiSeq2000 from Illumina (SR; 1 × 50 bp; ∼30–40 million reads per sample). Sequence images were transformed with Illumina software BaseCaller to bcl files, which were demultiplexed to fastq files with CASAVA v1.8.2. Quality check was done via fastqc; v0.10.0 (Andrews, 2010). Read alignment was performed using STAR; v2.3.0 (Dobin et al., 2013) to the mm10 reference genome with 2-pass mapping. Data was converted and sorted by samtools; v0.1.19 (Li et al., 2009) and reads per gene were counted via HTSeq; v0.5.4.p3 (Anders et al., 2015). Data analysis was performed using R/Bioconductor 3.0.2/2.12 (Luo and Brouwer, 2013); loading DESeq2 (Love et al., 2014). Sequence data will be deposited in NCBI’s Gene Expression Omnibus and are accessible through GEO Series upon acceptance of the manuscript.

### MeDIP Sequencing of FACS-Enriched tdTomato Cells

For genome-wide methylation analysis we applied immunoprecipitation methods for the enrichment of 5-methylcytosines. Specifically, 100 ng of genomic DNA were used as starting material. The Methylated-DNA IP Kit from Zymo (Cat. N° D5101) was applied according to manufacturer’s instructions. The product of the IP and control reaction were then used for preparation of Illumina compatible libraries according to the TruSeq Nano DNA Library Prep Kit (Cat. N° FC-121-4001). Libraries were sequenced on a HiSeq 2000 yielding 50 bp single end reads. The sequencing reads were demultiplexed using the Illumina CASAVA tool and sequence quality was checked using fastqc; v0.10.0 (Andrews, 2010). The reads were then aligned to the genome of *Mus musculus* (mm10) using Bowtie 2; v2.0.2 (Langmead and Salzberg, 2012). Briefly, reads were aligned using default parameters allowing for two mismatches using seed alignment. Differentially methylated regions (DMRs) were identified using the MEDIPS package for R; v1.16.0 (Lienhard et al., 2014) with a window size of 700 bp and a minimum coverage of 5% of the window length. Differential methylation analysis from low number of replicates was done using edgeR (Robinson et al., 2010) to estimate the biological variability and model the count data using negative binomial distribution. DMRs were considered gene-associated DMRs, or differentially methylated genes (DMGs), if they were inside a gene, in the promoter region [−1000, 0] of the transcription start site (TSS) or in the terminator region [0, +300] from the transcript termination site (TTS). DMRs were those with adjusted *P*-value <0.05. A detailed description of the analysis pipeline can be found in Halder et al. (2015). Sequence data will be deposited in NCBI’s Gene Expression Omnibus and are accessible through GEO Series upon acceptance of the manuscript.

### Integrative Analysis of FACS-Sorted Sequencing Data

Genes in the FACS RNA sequencing data were considered differentially expressed with a Benjamini-Hochberg adjusted *P* value *P* < 0.05 and a |logfc| > 1. Gene list overlaps between differentially expressed and methylated genes were quantified using the Jaccard coefficient. Absolute numbers of DMGs were determined without regard to multiple sites of differential methylation in a single gene. Significance of enrichment of methylated genes was calculated using Fisher’s exact test.

Gene lists including the genes showing both, differential methylation and expression, were submitted to the *Database for Annotation, Visualization and Integrated Discovery*^1^ (DAVID) for Gene Ontology (GO) or KEGG Pathway term enrichment analysis. Results of GO enrichment analysis were visualized in a bar diagram including the respective *Benjamini-Hochberg* corrected *P*-value, the number of genes and the enrichment fold change included in a certain term.

Heat maps were generated using R package pheatmap^2^. For heat maps showing comparison between two datasets, data was normalized to 6 months WT. In case of heat maps illustrating more than two samples, data was scaled. Significance levels: ^∗^*P* < 0.05; ^∗∗^*P* < 0.01; and ^∗∗∗^*P* < 0.001.

### Microscopy and Image Data Analysis

Images of immunohistochemistry staining of adult tissue sections or immunocytochemistry of stained cell culture was recorded either with an inverted confocal laser scanning microscope TCS SP5 (Leica Microsystems, Germany) or with an inverted transmitted light microscope Axio CellObserver Z1 equipped with MosaiX module for tile scanning and apotome for confocal like imaging (Carl Zeiss Microscopy, Germany). Photographs were analyzed using the free FIJI software (Schindelin et al., 2012).

For life cell imaging of CB cells transfected with the CD63-pEGFP and either control or *Dnmt1* siRNA, images were taken with Axio CellObserver Z1, ×40 optical magnification using apotome. Z-stack was applied over the whole cell and acquisition was performed every 5 min for 1 h. ^∗^.zvi-files were opened with FIJI; maximum intense projection was performed and data were exported as ^∗^.avi with five frames per second. The movement of CD63-pEGFP positive vesicles was measured direction specific from one timepoint to the next and speed was calculated based on the time interval. Analysis of cell number in adult sections was performed with ImageJ cell counter plugin. Counted cell numbers in section analysis were normalized to the area of the counted region.

For fluorescence intensity measurements, each experimental design was imaged at one particular microscope with consistent settings regarding exposure time and light intensity at the CellObserver Z1 or laser power, gain and spectral settings at the SP5 LSM. Fluorescence intensity measurement for the CD63 staining and LAMP1 staining was performed in the processes of the cells. For each picture, background correction was performed by subtracting the mean fluorescent intensity from three background areas. Mean fluorescent intensity of the *Dnmt1* siRNA treated cells was normalized to control siRNA. Photoshop CC was applied for image composition. Boxplots were plotted using R.

Significance was analyzed with two-tailed Student’s *t*-test or two-way ANOVA. Significance levels: ^∗^*P* < 0.05; ^∗∗^*P* < 0.01; and ^∗∗∗^*P* < 0.001.

## Results

### Vulnerability of PV-Expressing Neocortical GABAergic Interneurons Toward Aging

**FIGURE 1:**
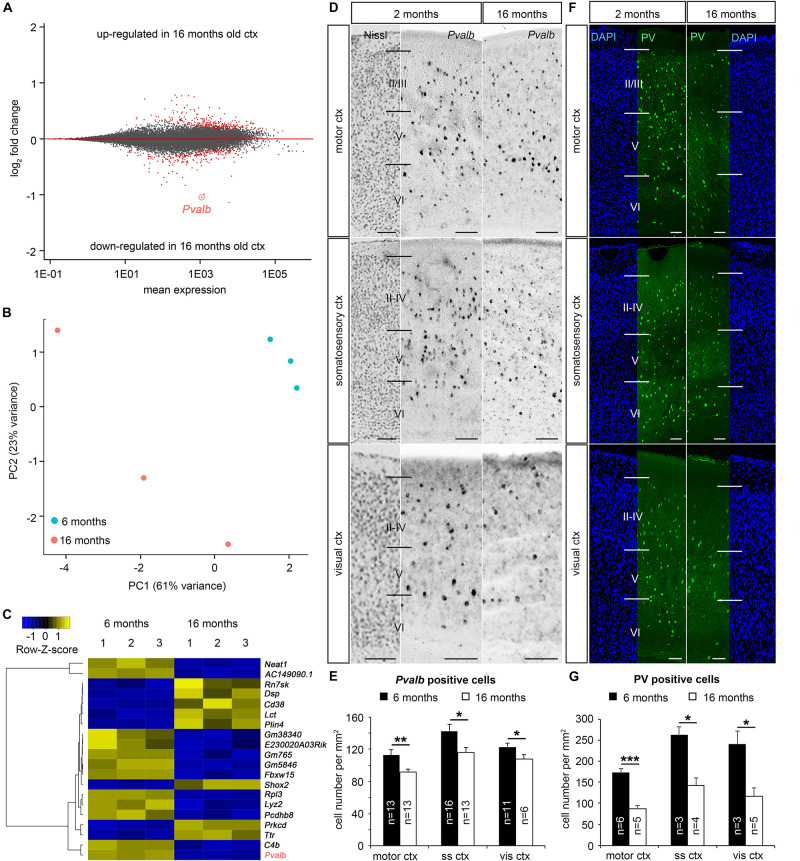
Age-dependent reduction of parvalbumin expression in the mouse cortex. **(A)** MA-plot illustrating differential gene expression between whole cortical tissue of 6 months (*n* = 3 brains) and 16 months old (*n* = 3 brains) C57BL/6 mice. RNA sequencing was performed to reveal age-dependent differential gene expression (red dots, *P* < 0.05, Benjamini adjusted). **(B)** Principal component analysis (PCA) illustrates the segregation of the different samples, whereby the first and second PC account for 61 and 23% of the variance, respectively. **(C)** Heat-map illustrating the 20 genes with highest absolute foldchanges among the differentially expressed genes between cortical tissue samples of 6 and 16 months old C57BL/6 WT mice including *Pvalb*, which is significantly reduced in aged samples. **(D–G)** *In situ* hybridization and immunohistochemistry against *Pvalb* mRNA **(D)** and PV protein **(F)** in the motor cortex, somatosensory cortex and visual cortex in 6 months and 16 months old C57BL/6 mice (*N* = 3 different brains per age), quantified in **(E)** and **(G)**, respectively (*P < 0.05; **P < 0.01; ***P < 0.001, *Student’s t-test*). Scale bars: 100 μm in **(D)** and **(F)**.

Aging-dependent functional defects in the cortical inhibitory GABAergic system were reported for humans (Cheng and Lin, 2013) as well as for different animal models (Miettinen et al., 1993; Ouda et al., 2008) including mice (Jessen et al., 2017). Since mice serve as key models to study the neurobiology of aging and age-associated neurodegenerative diseases (Jucker and Ingram, 1997; Bilkei-Gorzo, 2014), we tested whether the neocortical GABAergic system is compromised in aged mice. As an initial approach we performed differential gene expression analysis of the whole neocortex from young (6 months) and aged (16 months) C57BL/6 mice. In general, RNA sequencing revealed comparatively low numbers of age-dependent differentially expressed genes (DEG = 470 genes, Figure 1A), which additionally displayed small fold changes (ranging from −0.78 < log2fc < 1.14). This was also observed by others when using whole cortical tissue containing a mixed population of cells (e.g., glia versus neurons), which likely show different responses toward aging (Kimmel et al., 2019). In accordance with elevated inter-individual variability of gene expression observed in aged human brains (Kedlian et al., 2019), we also detected a similar variability in the cortical samples of aged mice (Figure 1B). These inter-individual differences heavily impact fold changes and differential gene expression analysis. Another hallmark of the aging brain is mRNA–protein decoupling (Wei et al., 2015), with numerous changes occurring mainly on the protein level (Liguz-Lecznar et al., 2015). However, as one of the most prominently differentially expressed genes *Pvalb* was identified, showing significantly diminished transcript levels in 16 months old cortex samples (adjusted *P* = 2.73E-50, log2fc = 1.04, Figures 1A,C), the time point when aging begins in mice (Xu et al., 2007). This finding was confirmed by *in situ* hybridization experiments, indicating an age-related reduction of *Pvalb*-expressing cells in motor, somatosensory and visual neocortical areas (Figures 1D,E). Consistently, we found less PV-immunoreactive cells (Figures 1F,G) in the same cortical regions in aged mice. Together, our data suggest a loss of PV-positive cortical interneurons in aged mice, being in line with the decrease of PV interneurons in somatosensory, auditory, and motor cortical areas of aged rats (Miettinen et al., 1993; Ouda et al., 2008).

In contrast to this depletion of inhibitory PV interneurons in the cerebral cortex, excitatory neurons, which account for >80% of cortical neurons (DeFelipe and Fariñas, 1992), appear less affected. Pan-neuronal density analysis of NeuN-positive cells did not reveal significant age-related changes (Supplementary Figure S1). In summary, we identified a vulnerability of PV-positive cortical inhibitory interneurons upon aging in mice.

### DNMT1 Affects the Long-Term Survival of Neocortical Interneurons

**FIGURE 2:**
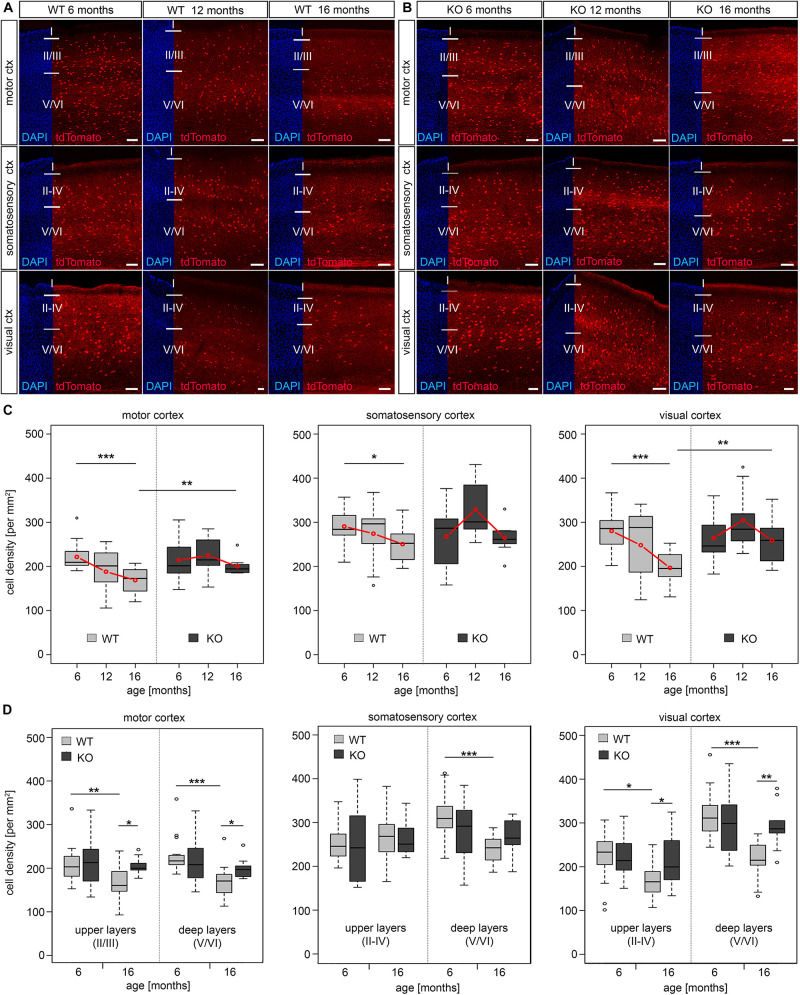
*Dnmt1* knockout enhanced long-term survival of cortical interneurons. **(A,B)** Microphotographs of sagittal sections (Bregma 1.44) illustrating the motor, somatosensory, and visual cortex of 6, 12 and 16 months old *Pvalb-Cre*/*tdTomato* (WT, **A**) and *Pvalb-Cre/tdTomato/Dnmt1 loxP*^2^ (KO, **B**) mice showing tdTomato (red) and DAPI positive cells (blue). The cell density per area is quantified in **(C)**, which revealed a significant loss of interneurons upon aging in *Dnmt1* WT (*P < 0.05, ***P < 0.001) for the motor, somatosensory and visual cortex, respectively (two-way ANOVA, Bonferroni corrected), but no significant age-dependent differences in *Dnmt1* KO mice. Comparison of aged genotypes revealed significant differences in the motor and visual cortex (**P < 0.01; *Student’s t-test*) **(D)** Layer-specific analysis of cell density in 6 and 16 months old *Dnmt1* WT and KO mice in the motor, somatosensory, and visual cortex (*P < 0.05, **P < 0.01; ***P < 0.001, *Student’s t-test*). The numbers of analyzed sections are listed as follows: 6 months old WT: *n* = 16, *n* = 15, and *n* = 13 for motor, somatosensory and visual cortex, respectively; 6 months old KO: *n* = 19 for motor cortex and *n* = 16 for somatosensory and visual cortex; 12 months old WT and KO: *n* = 9 sections for each cortical area; 16 months old WT: *n* = 14 for motor and visual cortex and *n* = 12 for somatosensory cortex; 16 months old KO: *n* = 9 for each cortical region (from *N* = 3 different brains per genotype and age). WT, wild-type; KO, knockout. Scale bars: 100 μm.

Changes of the epigenetic landscape by genomic methylation and histone modifications contribute to transcriptional control in aging and lifespan regulation (Zampieri et al., 2015). DNA methylation, executed by DNMTs, is a major epigenetic mechanism regulating gene expression in mammals during different stages of life (Johnson et al., 2012; Zampieri et al., 2015). DNMT1 is one of the main DNMTs expressed in the developing and adult brain. DNMT1 modulates neuronal survival (Hutnick et al., 2009; Feng et al., 2010; Pensold et al., 2017; Symmank and Zimmer, 2017) and synaptic function of both excitatory neurons (Meadows et al., 2015, 2016) as well as inhibitory interneurons (Pensold et al., 2020). Hence, we asked whether DNMT1 is involved in the regulation of cortical interneuron survival during aging. To this end, we exploited a mouse model described previously (Pensold et al., 2020), in which *Dnmt1* deletion is restricted to PV-cells (*Pvalb*-*Cre*/*tdTomato*/*Dnmt1 loxP^2^*). As controls, we used *Pvalb*-*Cre*/*tdTomato* mice. *Pvalb* promoter-dependent CRE recombinase-mediated *loxP* recombination drives persistent tdTomato protein expression, reported to start at the 5th week of life (Madisen et al., 2010). The analysis of the *Pvalb-Cre/tdTomato* interneuron density in adult versus aged mice confirmed the findings we obtained by RNA sequencing of whole cortical tissue, *in situ* hybridization, and immunostainings in C57BL/6 wildtype mice (Figure 1). We found a significant age-related reduction of tdTomato positive cells in motor and visual cortical areas of *Pvalb-Cre/tdTomato* mice (Figures 2A,C). Both superficial and deep cortical layers were affected by the reduction in interneurons (Figure 2D). Although less prominent, we also observed a significant decline of tdTomato cells in the somatosensory cortex (Figures 2A,C). This reduction was mainly restricted to the deep cortical layers (Figure 2D). At an intermediate stage (12 months old mice) we found a trend for reduced cell density, indicating that interneuron degeneration starts about one year of life (Figures 2A,C).

Next, we comparatively analyzed tdTomato cells in 6, 12, and 16 months old *Pvalb*-*Cre*/*tdTomato*/*Dnmt1 loxP^2^* mice in the motor, somatosensory and visual cortical areas. While in young mice no differences in interneuron numbers were observed compared to controls (Figures 2B,C), 16 months old *Dnmt1* KO mice maintained a significantly higher density of tdTomato positive interneurons in motor and visual cortical areas (Figures 2A–C). In the somatosensory cortex, we again observed a trend toward increased densities of *Dnmt1*-deficient interneurons compared to age-matched controls (Figures 2A,C). Hence *Dnmt1* deficiency substantially improves long-term survival of PV-expressing cortical interneurons, indicating that DNMT1 function either directly or indirectly impairs cortical PV-interneuron survival in aged mice. This is in striking contrast to DNMT1 function during brain development, where it promotes POA-derived interneuron survival through non-canonical actions (Pensold et al., 2017; Symmank et al., 2018, 2020).

### The Ameliorated Interneuron Survival in Aged *Dnmt1*-Deficient Mice Correlates With Improved Somatomotor Performances

**FIGURE 3:**
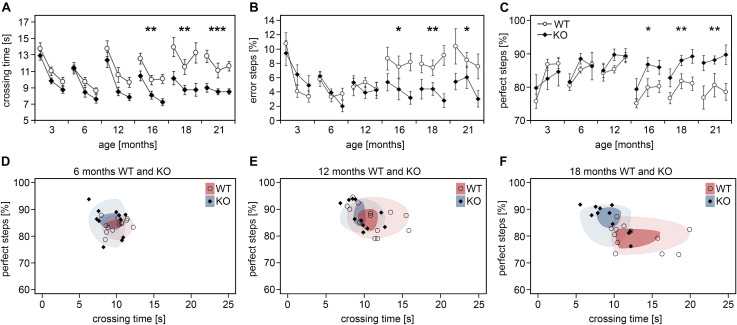
Aged *Pvalb*-*Cre*/*tdTomato*/*Dnmt1* KO mice show improved somatomotor performances. **(A–C)** Performance of *Pvalb*-*Cre*/*tdTomato*/*Dnmt1* KO (*N* = 10) and WT (*N* = 8) mice were tested in the inclined ladder rung test at distinct stages of life ranging from 3 to 21 months (three consecutive trials per stage). Crossing time **(A)**, error steps **(B)**, and perfect steps **(C)** were quantified (two-way ANOVA; data are shown as mean ± SEM; *P < 0.05, **P < 0.01, ***P < 0.001). **(D–F)** The scatter plots of perfect steps against crossing time for the distinct *Pvalb*-*Cre*/*tdTomato*/*Dnmt1* KO and WT mice at 6 months **(D)**, 12 months **(E)**, and 18 months **(F)** illustrate the stronger segregation of the cohorts with age shown by the decreasing overlap of the circles upon aging representing the 1st (dark colored) and 3rd quartile (light colored) of the data range per group.

Given their important role in cortical information processing, cortical interneuron decline was proposed to contribute to the cognitive and motoric impairments observed in the elderly (Bordner et al., 2011). To test whether attenuated interneuron loss correlates with improved skills in aged *Dnmt1*-deficient mice, we applied the ladder rung test to analyze motor performance that depends on somatomotor cortical activity (Metz and Whishaw, 2009). We continuously tested *Pvalb*-*Cre*/*tdTomato*/*Dnmt1 loxP^2^* and *Pvalb*-*Cre*/*tdTomato* mice at distinct stages of life ranging from 3 to 21 months. Consistent with observations of others (Hebert and Gerhardt, 1998) and the age-dependent changes in interneuron numbers, the motor performances of control mice deteriorated with age as determined by measuring the foot placement accuracy and crossing time (Figures 3A–C). In stark contrast, *Dnmt1*-deficient mice did not show corresponding age-related impairments for the parameters and the time course analyzed, hence performing significantly better than controls at 16 to 21 months of age (Figures 3A–C). When plotting the percentage of perfect steps against crossing time for KO and control mice at 6, 12, and 18 months (Figures 3D–F), cohort segregation increased with age.

In addition to cortical information processing, locomotion depends on cerebellar Purkinje cells and skeletal muscle function, tissues that also display *Pvalb* and *Dnmt1* expression (Supplementary Figures S2a,b; Racay et al., 2006). In skeletal muscle, DNMT1 indeed plays a role during differentiation and regeneration (Aguirre-Arteta et al., 2000; Wang et al., 2015). However, neither in skeletal muscle nor in the cerebellum, obvious abnormalities were observed upon *Dnmt1* deletion. Purkinje cell numbers in the cerebellum were not affected by PV-CRE mediated *Dnmt1* deletion, neither in the young nor in the aged mice (Supplementary Figures S2c–e). Moreover, muscle integrity, structure, and innervation were not altered by *Dnmt1*-deletion at the stages investigated, as determined by hematoxylin/eosin, laminin and neuromuscular junction staining, respectively (Supplementary Figures S2f–k). These data strongly suggest that the motor impairments in aged controls are caused by the loss of cortical interneurons, which can be attenuated by *Dnmt1* deletion.

### PV Interneurons Show an Increase in Degradation- and a Decline in Synapse-Related Gene Expression Upon Aging

Highlighting age-mediated transcriptional changes might help to approach the underlying mechanisms of the DNMT1-dependent PV-interneuron loss. This requires enrichment of PV-positive cortical interneurons from adult versus aged brains, as these interneurons represent a minority of the neocortical neuronal population (Druga, 2009). To this end, we applied an optimized protocol for adult cortical neuron isolation applicable for FACS. We combined mechanical and trypsin/collagenase-based enzymatic dissociation with trehalose treatment and *Percoll* density gradient centrifugation, as described and validated recently (Pensold et al., 2020).

**FIGURE 4:**
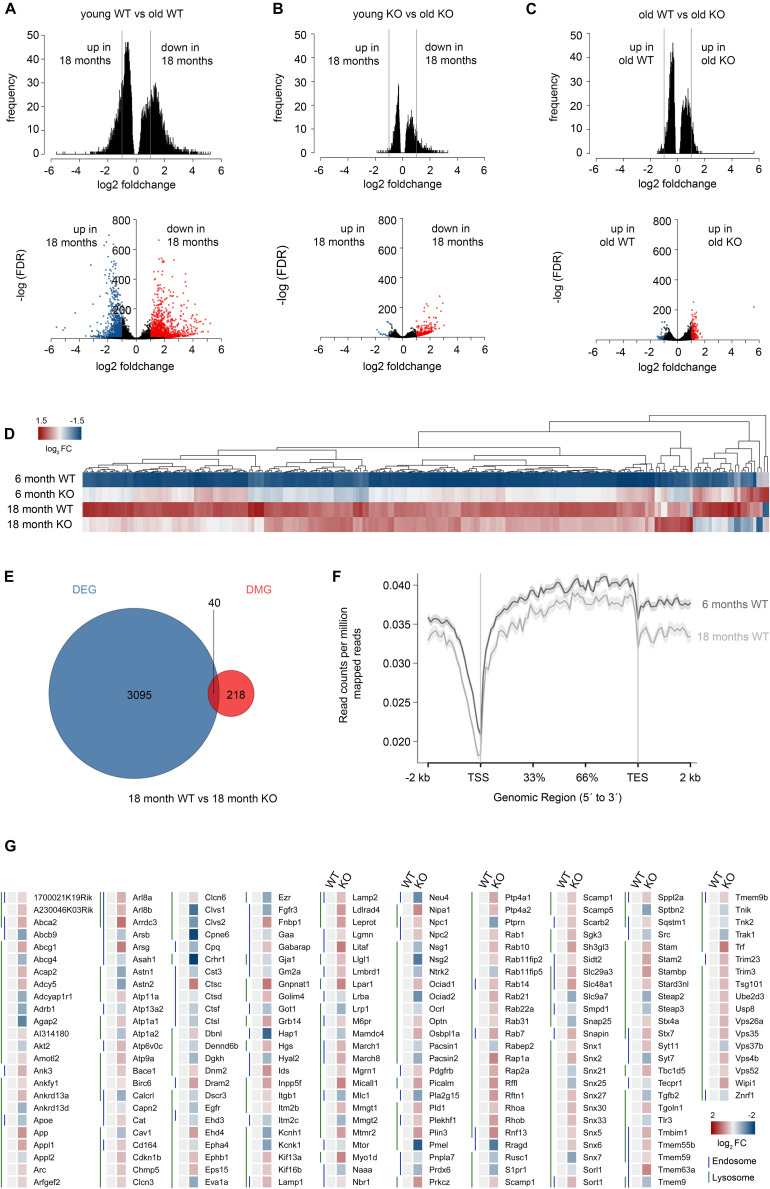
Correlative transcriptome and methylation analysis of adult and aged *Dnmt1*-deficient and wild-type *Pvalb*-expressing cortical interneurons. **(A–C)** Density plots (upper panel) and volcano plots (lower panel) illustrating significant changes in gene expression determined between FACS-enriched young and old *Dnmt1* WT interneurons **(A)**, young and old *Dnmt1* knockout (KO) cortical interneurons **(B)**, as well as between old WT and KO interneuron samples (**C**; *P* < 0.05, Benjamini adjusted; pooled samples from *N* = 6 WT and KO mice at 6 months; and *N* = 9 WT and *N* = 12 KO mice at 18 months analyzed in technical duplicates). Blue and red-colored dots in the volcano plots represent genes with |logfc| > 1. **(D)** Heat-map illustrating the re-scaled expression of genes in all samples, which were found differentially expressed between young (6 months) KO and young WT interneurons. **(E)** Venn diagram illustrating the overlap (*P* = 3.388E-5, *Fisher’s Exact test*) of differentially expressed genes (DEG) and differentially methylated genes (DMG) between aged FACS-enriched *Pvalb*-*Cre*/*tdTomato*/*Dnmt1* WT and KO cortical interneurons as determined by RNA-sequencing (*P* < 0.05, Benjamini-adjusted) and MeDIP-sequencing (*n* = 9 WT and *n* = 12 KO mice; *P* < 0.05, Benjamini-adjusted). **(F)** Methylation plot illustrating the average DNA methylation levels of a random sample of 10% of the genes from the *mm10* reference genome in young (6 months) and old (18 months) cortical interneurons from *Dnmt1* WT mice. **(G)** Heat-map of differentially expressed genes associated to the GO terms *endosome* and *lysosome*, normalized to 6 months old WT.

Previously, Xu et al. (2007) investigated murine brain tissue at 6, 16 and 24 months of age, and found that most age-dependent genes are not differentially expressed at the age of 16 months. Hence, we chose to analyze interneurons of 18 months old control versus conditional *Dnmt1* knockout mice to monitor an advanced stage of aging, and compare interneuron transcriptional profiles with 6 months old mice for each genotype. Consistent with the PV interneuron loss in aged controls, we revealed significantly reduced FACS-events per hemisphere for aged *Pvalb*-*Cre*/*tdTomato* mice compared to the 6 months old mice (Supplementary Figure S3a). Transcriptome comparison between FAC-sorted young and old control interneurons illustrated that aging is associated with prominent changes in gene expression (Figure 4A and Supplementary Figures S3b–d). A total of 3,384 genes were differentially regulated (adjusted *P* < 0.05, |logfc| > 1), of which 65% were down-regulated and 35% up-regulated with age (Figure 4A). This high number of age-dependent transcriptional changes exceeds the transcriptional alterations revealed for the whole cortex (Figures 1A,B), which captures different aging signatures of diverse cell types collected in the cortical samples (Stegeman and Weake, 2017; Kimmel et al., 2019).

Among the genes we found to be up-regulated upon aging, GO-enrichment analysis revealed significantly enriched transcripts related to *membrane*, *endoplasmatic reticulum*, *endosome*, and *exosome* (Supplementary Table S1). The up-regulation of endosome and exosome-related genes in cortical interneurons might reflect an elevation of degradative actions and mechanisms upon aging in response to the accumulation of defective proteins, to maintain neuronal functionality over life time.

Of note, functional impairment of exosomes in transferring proteins, mRNAs, and miRNAs has been related to synaptopathies (Pitt et al., 2017), and synaptic dysfunction is considered a hallmark in neuronal aging (Deak and Sonntag, 2012; Azpurua and Eaton, 2015) and neurodegenerative disorders (Freeman and Mallucci, 2016; Ghiglieri et al., 2018). Altered or impaired synaptic function of aged PV-expressing interneurons is strongly supported by the profile of genes that were down-regulated upon aging. By GO analysis, synapse-related genes were detected as most significantly overrepresented, displaying by far the highest enrichment scores (Benjamini-adjusted *P* = 1.91E-61; FDR = 4.6E-61; Supplementary Table S1). Moreover, genes collected in the GO-terms *membrane*, *cell junction*, *plasma membrane*, *dendrite* and diverse ion transport and ion channel-related genes were strongly enriched among the genes determined as transcriptionally down-regulated in aged wild-type interneurons (Supplementary Table S1). Of note, we have not identified a significant enrichment of cell death or survival associated genes among the genes changed in expression between young and old interneurons (Supplementary Table S1). In sum, the transcriptional alterations that we detected in aged neocortical PV-positive interneurons suggest an age-related impairment of synaptic functionality. Moreover, alterations in the degradation machinery can be assumed from the transcriptional alterations, which can influence neuronal survival (Kim and Seo, 2014).

### *Dnmt1* Deficient PV Interneurons Display Diminished Age-Associated Transcriptional Alterations

In addition to ameliorated locomotion, the attenuated decline in interneuron density in aged *Dnmt1* knockout mice coincides with diminished age-associated quantitative transcriptional changes in *Pvalb*-*Cre*/*tdTomato*/*Dnmt1 loxP^2^* interneurons (Figure 4B; Supplementary Table S2). Compared to control interneurons, aging in *Pvalb*-*Cre*/*tdTomato*/*Dnmt1 loxP^2^* cortical interneurons was characterized by both fewer differentially expressed genes and decreased fold changes. Only 383 genes were differentially expressed (adjusted *P* < 0.05, |logfc| > 1, Figures 4A,B). For better illustration of the discrete changes in expression between all samples, we re-scaled the expression levels of genes relative to the expression range of all groups (young and old control as well as knockout samples; Figure 4D). The heatmap shown in Figure 4D depicts prominent age-related transcriptional alterations in controls, but rather mild alterations in *Dnmt1*-deficient interneurons. These data are consistent with the attenuated age-associated decline observed for conditional *Dnmt1*-knockout mice at cellular and behavioral level.

A common denominator of age-mediated transcriptional remodeling in both genotypes is that age-related down-regulation dominates over up-regulation for the significantly altered genes with |logfc| > 1 (Figures 4A,B). For age-associated gene expression changes in *Pvalb*-*Cre*/*tdTomato*/*Dnmt1 loxP^2^* interneurons, about 96% of differentially expressed genes were down-regulated (Figure 4B). Consistently, a “shutdown” of transcription in the aged cortex has been described before (Xu et al., 2007). Another similarity between aging control and *Dnmt1*-knockout interneurons was a significant enrichment of down-regulated synapse-related genes (Supplementary Tables S1, S2).

### Potential Implication of DNA Methylation in Age-Mediated Transcriptional Remodeling

DNA methylation was frequently proposed to contribute to the aging-associated transcriptional changes (Issa, 2002; Jones et al., 2015). To this end, we conducted differential methylation analysis by MeDIP-sequencing of FAC-sorted interneurons from young (6 months) and aged (18 months) control mice to monitor age-related alterations in DNA methylation levels. Further, we determined genes whose age-related transcriptional changes (adjusted *P* < 0.05) correlated with alterations in the DNA methylation level (adjusted *P* < 0.05). Among the 201 genes which demonstrated changes in expression and DNA methylation upon aging, *synapse*, *cytoskeleton*, *dendrite*, *postsynaptic density*, and *membrane*-related genes were significantly overrepresented (Supplementary Table S3), indicating that DNA methylation is implicated in the age-related transcriptional changes of these genes.

To determine which genes are differentially expressed and methylated in aged interneurons in a DNMT1-dependent manner, we first compared transcriptional profiles and DNA methylation signatures of old control and *Dnmt1*-deficient interneuron samples. We obtained only 258 differentially expressed genes (adjusted *P* < 0.05) displaying a |logfc| > 1 (Figure 4C). A similar number of 218 genes showed differential methylation (adjusted *P* < 0.05). However only two of these DMGs were overlapping with the pool of differentially expressed genes. Hence, we included all significantly differentially expressed genes independent of their fold change (3,095 genes) for correlation analysis between changes in methylation and transcription. Only 40 genes were significantly changed in both expression and methylation between the aged genotypes (Figure 4E). This indicates that DNMT1-dependent DNA methylation might play a rather minor role for the transcriptional changes, once the interneurons reach the age of 18 months. Indeed, the efficiency of the catalytic activity of DNMT1 is described to be reduced in an age-dependent manner (Casillas et al., 2003). This is in line with the global reduction of DNA methylation levels observed upon aging in control interneurons with MeDIP sequencing (Figure 4F), a finding that corroborates the age-related global hypomethylation reported by others (Shimoda et al., 2014; Lardenoije et al., 2015).

For those 40 genes (Figure 4E) which simultaneously changed in both expression and DNA methylation between aged genotypes, GO analysis revealed a significant enrichment of *actin cytoskeleton* and *postsynaptic density*-related genes, which are putatively regulated by DNMT1-dependent DNA methylation even in interneurons of advanced age (Supplementary Table S4). This fits to our finding that synapse and cytoskeleton-related genes are DNA methylation-dependently changed in expression upon aging in control cells (Supplementary Table S3). Albeit MeDIP sequencing covers only 15–16% of total 5-mC content (Stirzaker et al., 2014), for which the data provide only limited information, having a closer look on DNMT1 target genes identified in younger mice might provide further insights in the causes of impaired long-term interneuron survival.

### DNMT1-Dependent DNA Methylation in Adult Interneurons Affects Degradative Pathways

In stark contrast to the comparison of the 18 months old genotypes, we determined a highly significant overlap (*P* = 2.2E-16, *Fisher’s Exact test* for gene set enrichment analysis; odds ratio = 0.434) of 645 genes between young control and *Dnmt1* knockout interneurons, which display significant differences in both DNA methylation and gene expression (Pensold et al., 2020).

In general, far more genes were differentially expressed (3,868 genes) and/or methylated (3,135 genes) between young genotypes (Pensold et al., 2020). However, among neither the differentially expressed genes, nor among those genes both differentially expressed and methylated, we found a significant enrichment of apoptosis or cell death-related genes (data not shown). Hence, in contrast to developing interneurons, in which DNMT1 regulates expression of apoptosis genes (Pensold et al., 2017), survival regulation of interneurons in the aged cortex seems to result from different actions and targets of DNMT1.

**FIGURE 5:**
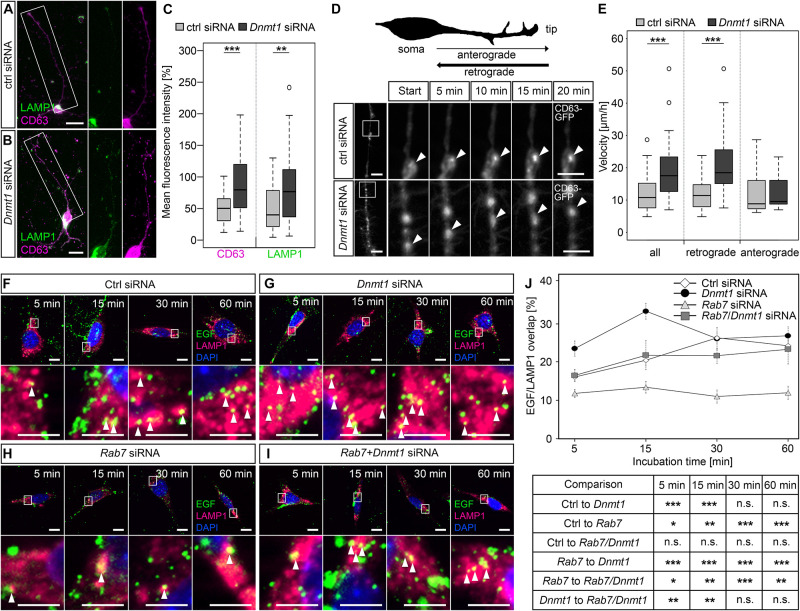
DNMT1 regulates retrograde transport of endosomes and endocytic-based degradation. **(A,B)** CD63 (magenta) and LAMP1 (green) antibody staining in MGE cells (E15 + 7 div) 24 h after control and *Dnmt1* siRNA treatment. The white rectangles in **(A,B)** illustrate the locations of the magnified parts of the processes. Quantification of fluorescence intensities is shown in **(C)**. **(D,E)** Cerebellar granule (CB) cells were co-transfected either with control or *Dnmt1* siRNA and a CD63-GFP expression plasmid, and the movement of CD63-GFP positive structures was imaged for 20 min. The location of the highly magnified sections along the neurite-like processes are illustrated by white squares in the low magnifications. **(D)** Schematic illustration of the morphology of a cultured cerebellar granule (CB) cell. The quantification is depicted in **(E)**. **(F–J)** The EGF-degradation assay with CB cells either transfected with **(F)** control siRNA, **(G)** *Dnmt1* siRNA, **(H)** *Rab7* siRNA, or **(I)** *Dnmt1* and *Rab7* siRNA. The co-localization of Alexa488-labeled EGF (green) and LAMP1 positive lysosomal structures (red) was quantified after 5, 15, 30, and 60 min, as quantified in **(J)**. CB cells, cerebellar granule cells; N2a cells, neuroblastoma cells. *Student’s t-test* in **(C,E,J)** with *P < 0.05, **P < 0.01, and ***P < 0.001. Scale bars: 20 μm in **(A,B)**; 10 μm in **(D, F–I)**; 5 μm in magnified sections in **(D,F–I)**.

Among the genes which we identified as repressed by DNMT1-dependent DNA methylation in young controls, we found an overrepresentation of *endocytosis* and *endosome*-related genes (Pensold et al., 2020). Furthermore, during analysis of all genes differentially expressed upon *Dnmt1* deletion, irrespective of altered DNA methylation, *lysosome* and *ubiquitination*-related genes were also found repressed by DNMT1 (Pensold et al., 2020; Figure 4G). Together these results demonstrate that endocytosis and degradative pathways are controlled by DNMT1. In a previous study we confirmed that dynamic DNMT1-dependent DNA methylation regulates synaptic transmission through the modulation of endocytosis-mediated vesicle recycling, which was improved upon *Dnmt1* deletion (Pensold et al., 2020). Hence, elevated GABAergic transmission and synaptic activity could indirectly promote interneuron survival of *Dnmt1*-deficient interneurons upon aging. However, endocytosis and endosomal function are crucial not only for synaptic activity regulation, but also affect degradative pathways (Ehlers, 2000; Gruenberg, 2001). Consistent with the transcriptional changes in 6 months old *Dnmt1*-deficient cortical interneurons (Figure 4G), siRNA-mediated *Dnmt1* depletion (knockdown efficiency of *Dnmt1* siRNA is illustrated in Supplementary Figure S4a) caused augmented CD63 and LAMP1 immunoreactivity, labeling endosomal, and lysosomal structures, respectively. This was determined in neurites of interneurons prepared from the embryonic MGE (Figures 5A–C) that give rise to PV interneurons, as well as in neurite-like processes of CB cells and neuroblastoma N2a cells 24 hours after transfection (Supplementary Figures S4b–d).

Endosomal-based degradation involves ubiquitination, retrograde transport to the cell soma, and fusion with lysosomes (McMahon and Boucrot, 2011; Haglund and Dikic, 2012). To quantify whether retrograde shuttling of endosomal compartments is influenced by DNMT1, we transfected CB cells with a CD63-GFP construct and analyzed transport velocity in the neurite-like processes upon *Dnmt1* knockdown and control siRNA transfections. While anterograde transportation was not changed in speed, we determined significantly faster velocities for retrograde transport of CD63-GFP particles after *Dnmt1* depletion (Figures 5D,E; Supplementary Movies 1, 2).

As the binding of EGF to epidermal growth factor receptors (EGFR) induces their internalization and degradation via the endo-lysosomal pathway (Haglund and Dikic, 2012), we next applied Alexa488-coupled EGF to CB cells and monitored the co-localization of EGF with LAMP1-positive lysosomal compartments at different time points. Indeed, siRNA-mediated *Dnmt1* depletion caused increased co-localization of EGF with LAMP1-positive lysosomes, both 5 and 15 min after EGF application (Figures 5F,G,J), indicating transport to lysosomal compartments. With longer incubation times, no differences to control siRNA-treated cells were observed (Figures 5F,G,J).

Lysosomal trafficking of the EGF-EGFR complex depends on RAB7, which mediates the fusion of late endosomes with lysosomes (Bucci et al., 2000). Consistently, we revealed a reduced EGF/LAMP1 co-localization after *Rab7* siRNA transfection of CB cells at all-time points tested (Figures 5H,J). *Rab7* expression was significantly up-regulated in *Dnmt1*-deficient PV-positive cortical interneurons (Figure 4G), and shown to be regulated by DNMT1-dependent DNA methylation (Pensold et al., 2020). Thus, we additionally analyzed the EGF/LAMP1 co-localization in *Dnmt1* siRNA-treated CB cells that were co-transfected with *Rab7* siRNA (knockdown efficiency of *Rab7* siRNA is depicted in Supplementary Figure S4a) to counteract the gain in *Rab7* expression in *Dnmt1*-siRNA transfected cells. This reversed the *Dnmt1* siRNA-triggered increase in EGF/LAMP1 co-localization (Figures 5I,J), suggesting that DNMT1 restricts endocytic-based degradation partly through repression of *Rab7* expression.

Ubiquitination is a common denominator in the targeting of substrates to the main protein degradation pathways (Clague and Urbé, 2010), including lysosomal degradation (reviewed in Clague and Urbé, 2006). Interestingly, we determined elevated proportions of ubiquitin-positive cortical interneurons evident in *Dnmt1*-deficient mice (50 ± 0.8%) compared to wild-type controls (39.5 ± 2%; ^∗∗^*P* < 0.01, *Student*’s *t*-test; *n* = 3 mice per genotype; Supplementary Figures S4e,f). Together, our data indicate that DNMT1 acts repressive on intracellular degradative pathways, which could affect long-term neuronal survival.

## Discussion

We here provided evidence that DNMT1 is implicated in the compromised long-term survival of inhibitory PV interneurons in the murine cerebral cortex. Aging is characterized by reduced PV interneuron numbers accompanied by declined somatomotor performance and prominent transcriptional remodeling. All effects were attenuated by *Dnmt1* deletion in PV interneurons. While DNMT1 promotes neuronal survival in the developing nervous system, it seems to compromise the long-term survival of PV-interneurons in the aged cortex. However, global transcriptome analyses did not point to a DNMT1-dependent transcriptional regulation of survival or cell death related genes causing the age-related interneuron loss. As repressive DNMT1-dependent DNA methylation restricts synaptic transmission as well as degradative pathways in adult PV interneurons, we hypothesize that impaired long-term survival is an indirect consequence of DNMT1-mediated modulation of synaptic activity and degradation over life-time.

Besides reduced excitability and plasticity (Clark and Taylor, 2011) and declined inhibitory function (Shetty and Turner, 1998; Stanley and Shetty, 2004; Cheng and Lin, 2013), a selective vulnerability of particular neuronal subtypes, like inhibitory interneurons, and GABAergic synapses (Rozycka and Liguz-Lecznar, 2017) was reported in the context of brain aging. Indeed, given the crucial role GABAergic inhibitory interneurons have in cortical information processing, age-dependent defects in inhibitory circuits provide an attractive hypothesis for cognitive decline and age-associated disorders (Rozycka and Liguz-Lecznar, 2017).

Our finding of reduced PV interneuron numbers in old cortices confirms previous studies, that reported a decline in SOM-, CB-, VIP-, and NPY-positive interneurons across species and brain regions (reviewed in Zimmer-Bensch, 2019a). Surprisingly, DNMT1 is implicated in the age-related PV interneuron loss.

Physiological aging involves a decline in synaptic density and functionality (Tanaka et al., 1996; Burke and Barnes, 2006; Polydoro et al., 2009; Berchtold et al., 2013), which includes inhibitory cortical synapses in the cerebral cortex (Rozycka and Liguz-Lecznar, 2017; Calì et al., 2018). Accordingly, aged control mice revealed synapse-related gene downregulation in PV interneurons (Supplementary Table S1), which correlated with altered DNA methylation (Supplementary Table S3). Similarly, others reported major changes in neurotransmission-related gene expression and repression of GABA-related transcripts in the human prefrontal cortex (Loerch et al., 2008) and across different species (reviewed in Rozycka and Liguz-Lecznar, 2017; Zimmer-Bensch, 2019a).

Some age-regulated synapse-related genes appear to be subject to DNMT1-dependent DNA methylation (Supplementary Table S4). Thus, we propose an age- and DNMT1-dependent shutdown of synapse-associated gene expression, which impairs synaptic function. As activity-dependent signaling is described to boost neuronal health through diverse mechanisms, decreased synaptic functionality could affect neuronal survival. Besides transcriptional control of pro- and anti-apoptotic genes, availability of neurotrophic factors and elevation of antioxidant defenses are modulated by neuronal activity (reviewed in Bell and Hardingham, 2011).

We have recently shown that DNMT1 acts on synaptic function of cortical PV interneurons in young mice, modulating GABAergic transmission (Pensold et al., 2020). Alterations in transmitter release affect synaptic strength and both are decreased upon aging (Kumar et al., 2007). Hence, it is conceivable that increased synaptic transmission rates in young *Dnmt1*-deficient interneurons exert protective effects on age-associated synaptic impairments, thereby indirectly promoting survival in aged *Dnmt1*-deficient mice.

Indeed, despite reports of DNMT-dependent developmental regulation of neuronal survival (Hutnick et al., 2009; Rhee et al., 2012; Pensold et al., 2017), direct evidence in the aging brain is still lacking. Comparing gene expression among PV interneuron populations, we found no evidence – in contrast to developing interneurons – that DNMT1 does affect long-term survival in aging brains by transcriptional control of survival- and/or cell death-related genes. Albeit, profiled at high resolution, we did not detect significant expression changes of cell survival or death-associated genes, neither among young and aged controls, nor when comparing *Dnmt1*-deficient and control interneurons. The same is true for genes which were both changed in methylation and transcription upon aging in controls, or between the genotypes, indicating that DNMT1-dependent DNA methylation modulates other processes, which then indirectly affect interneuron survival. Yet, MeDIP sequencing was reported to provide only a limited picture and resolution, e.g., compared to whole genome bisulfide sequencing (Stirzaker et al., 2014). For this our methylation analysis should be interpreted with caution and does not claim to provide an exhaustive picture. What we can state is that our analytical pipeline revealed DNMT1- and age-dependent changes in expression and methylation of proteostasis associated genes, which is supported by functionally validation studies.

Of note, long-term neuronal health ultimately depends on the proteostasis network. Age-related decline in protein homeostasis can cause diverse cellular dysfunctions, contributing to numerous neurodegenerative disorders (Douglas and Dillin, 2010). Endosome-based degradative pathways are crucial for processing and removing defective proteins or protein aggregates by proteolytic degradation in lysosomes (McMahon and Boucrot, 2011). Lysosomes digest both intra- and extracellular material after autophagy or endocytosis, respectively (Stoka et al., 2016). Lysosomal degradation is compromised in aged neurons (reviewed in Loeffler, 2019), and lysosomal dysfunction is associated with aging and numerous neurodegenerative disorders (Jiang et al., 2001), including Parkinson’s and Alzheimer’s disease (Büttner et al., 2013; McBrayer and Nixon, 2013; Wolfe et al., 2013; Menezes et al., 2015).

Lysosome-dependent lifespan regulation relies on their fundamental role in autophagy, which reportedly influences longevity. Mice lacking *Atg7* (autophagy related 7), encoding for the E1-like activating enzyme, that is essential for autophagy (Komatsu et al., 2005), develop neuronal loss and die within 28 weeks (Komatsu et al., 2006). In addition, suppression or loss of autophagy in the central nervous system causes neurodegenerative disease in mice (Hara et al., 2006; Komatsu et al., 2006), illustrating the relevance of the proteostasis network for neuronal survival.

A declining proteostasis network accompanies aging and triggers ineffective protein degradation. Aggregation of defective proteins, in turn, eventually leads to cell death (Douglas and Dillin, 2010). Hence, up-regulation of proteostasis-related genes in control interneurons indicates a compensatory response of aging neurons to counteract the remittent proteostasis network (Douglas and Dillin, 2010). This corroborates a previous report of age-related increases in LAMP-2a and HSPA8/Hsc70 concentrations in the mouse retina (Rodríguez-Muela et al., 2013), suggested to compensate for an age-related decrease in macroautophagy. Age-dependent HSPA8/hsc70 elevation was also seen in hippocampus, cortex, cerebellum, septum, and striatum (Calabrese et al., 2004).

Interestingly, such increase in proteostasis-associated gene expression was not detected upon aging in *Dnmt1*-deficient interneurons. This can be explained by the finding that *Dnmt1* deletion itself acts on proteostasis-associated gene expression in young interneurons. Compared to equal-aged controls, *endocytosis*-, *endosome*-, and *lysosome*-related gene expression was augmented in *Dnmt1*-deficient samples (Pensold et al., 2020, Figure 4G). While we previously verified that endocytosis-mediated elevated vesicle recycling increases GABAergic transmission of *Dnmt1*-deficient interneurons (Pensold et al., 2020), DNMT1-dependent regulation of degradative pathways so far remained unattended. Here, we validate that *Dnmt1* depletion elevates retrograde endosomal transport and lysosomal targeting, pointing to an improved degradative machinery upon *Dnmt1* depletion. Such boosted degradative actions could be neuroprotective or beneficial for neuronal survival in the long run, preventing age-related interneuron loss as seen in *Dnmt1*-deficient mice.

Together, our data suggest that dysregulation of cell death and/or survival related genes by DNMT1-dependent actions appears to play, if at all, a rather minor role as a potential mechanism underlying the age-related interneuron loss. We anticipate that DNMT1-dependent changes in aged interneurons result from cumulative effects of DNMT1 function during life-time, as the enzyme modulates two crucial aspects of neuronal function: synaptic activity and proteostasis. Hence, we propose a scenario, in which *Dnmt1* deficiency-induced enhancement of synaptic and/or proteostasis function in PV interneurons prevents or delays the age-related degeneration of these cells.

## Data Availability Statement

The datasets generated for this study can be found in the GEO database [Series GSE145026].

## Ethics Statement

The animal study was reviewed and approved by Thüringer Landesamt, Bad Langensalza, Germany.

## Author Contributions

AH performed experiments, data analysis, design of data analysis, figure illustration, and assisted in writing the manuscript. DP, JT, and JG designed and performed experiments, data analysis, and figure illustration. CB performed experiments, data analysis, figure illustration, and assisted in writing the manuscript. LG-B performed experiments, data analysis, and figure illustration. JL performed experiments, figure illustration, and manuscript correction. TP provided help with conceptual design and discussion of results. TL data analysis and design of data analysis. GS-R performed experiments. LM-B performed experiments and data analysis. JM and AU designed and performed experiments, data analysis, and manuscript correction. MS conceptual design and assisted in writing the manuscript. GZ-B conceptual design of the study, designed and performed experiments, data analysis, figure illustration, and wrote the manuscript. All authors contributed to the article and approved the submitted version.

## Conflict of Interest

The authors declare that the research was conducted in the absence of any commercial or financial relationships that could be construed as a potential conflict of interest.

## Footnotes

1 https://david.ncifcrf.gov

2 https://CRAN.R-project.org/package=pheatmap
