# Supplementary figures and images for: DNA Methyltransferase 1 (DNMT1) Function Is Implicated in the Age-Related Loss of Cortical Interneurons
Source: Front Cell Dev Biol. 2020 Jul 22;8:639. doi: 10.3389/fcell.2020.00639 (PMC7387673; doi:10.3389/fcell.2020.00639)

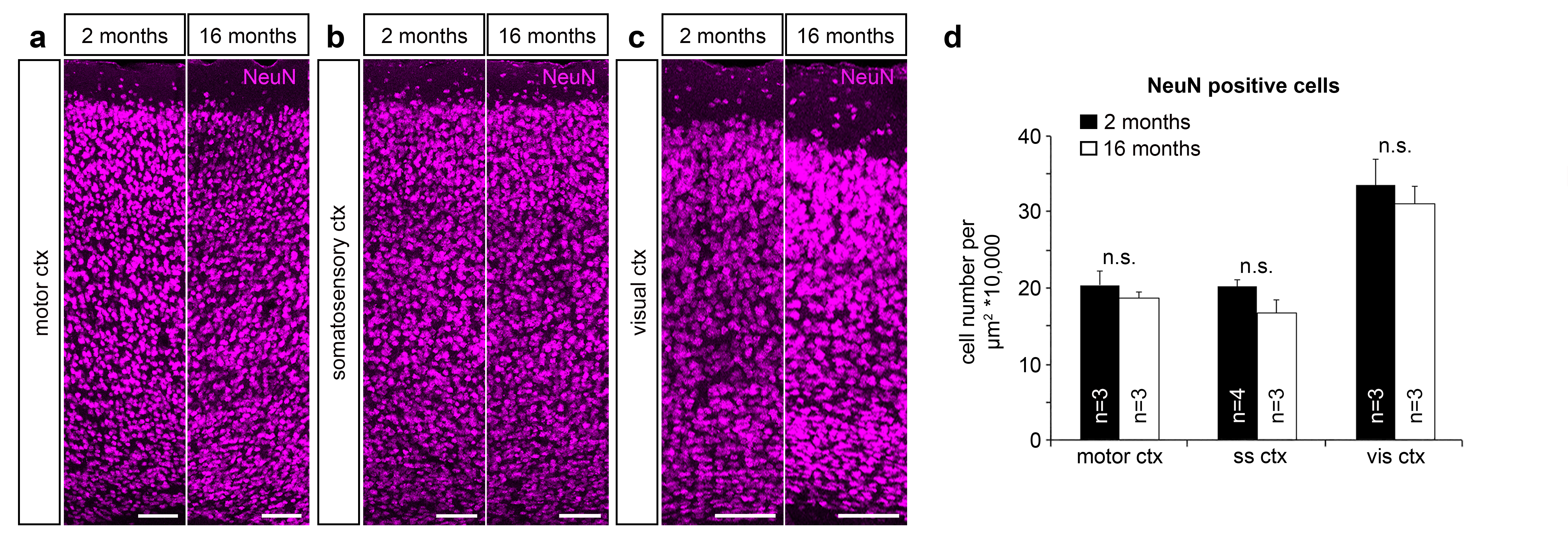

Supplement: Supplementary file 2 [file Image_1.tif]

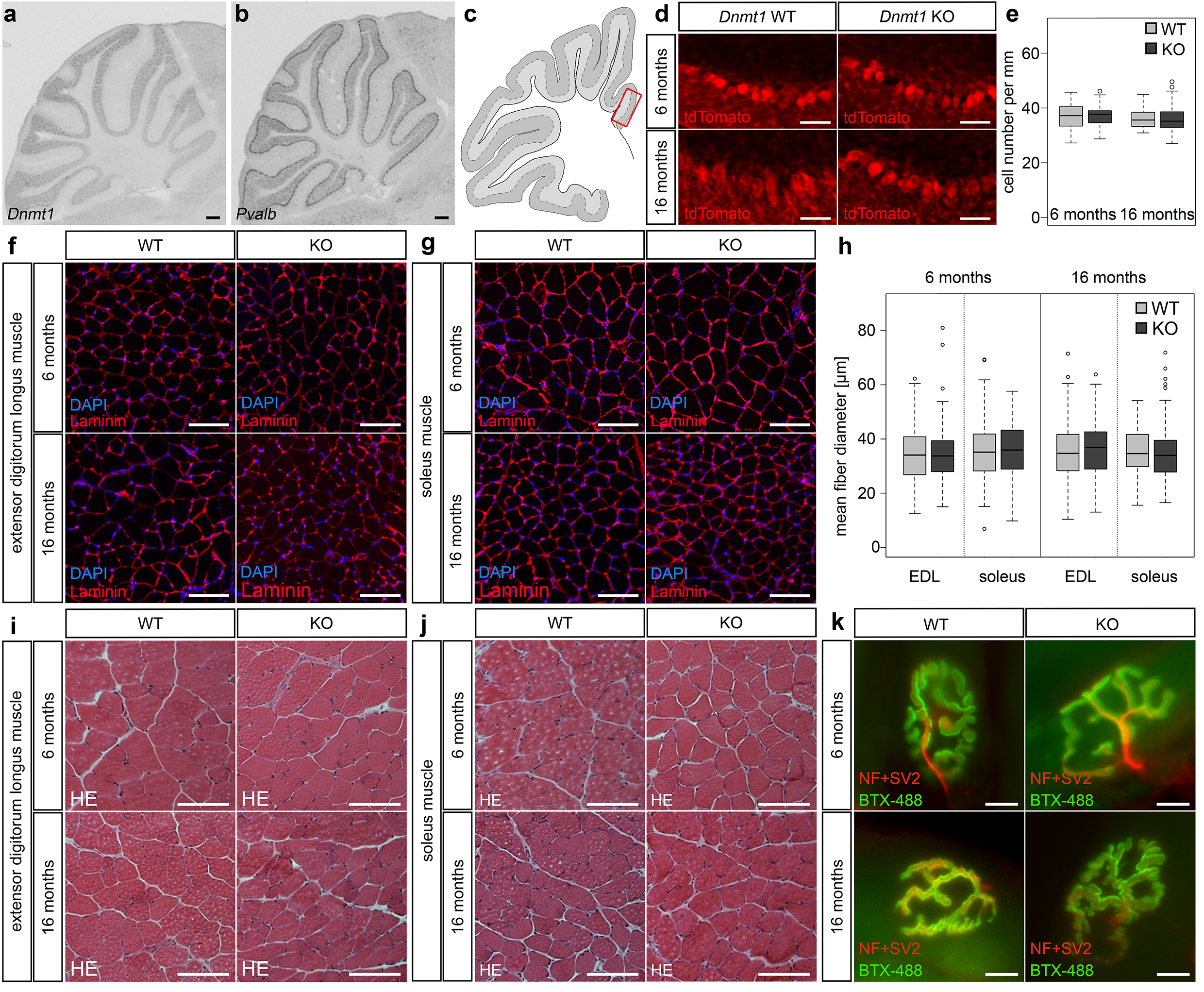

Supplement: Supplementary file 3 [file Image_2.tif]

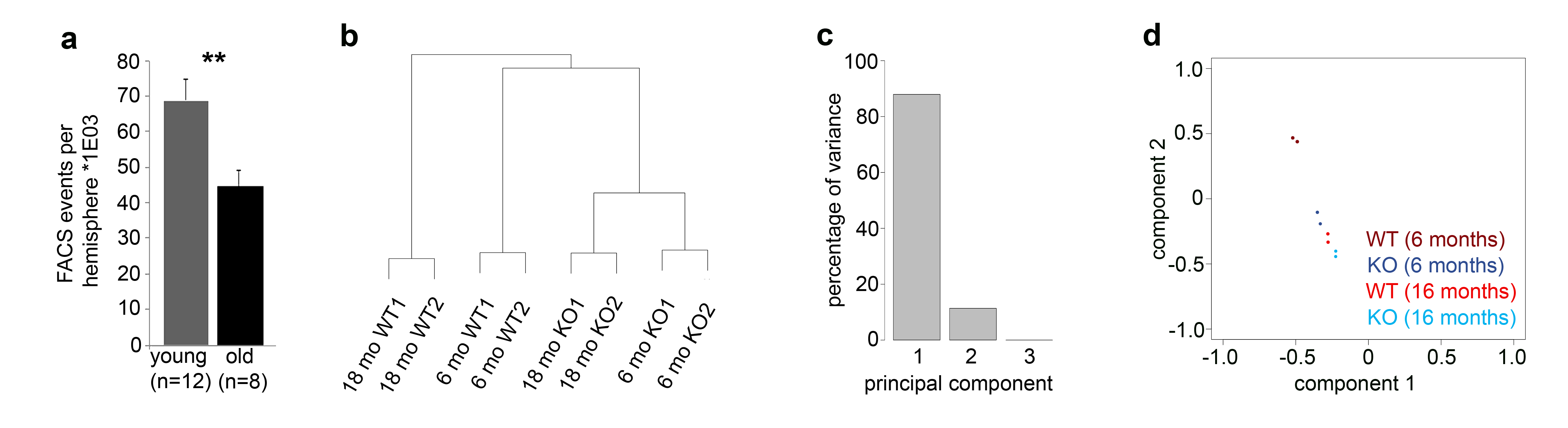

Supplement: Supplementary file 4 [file Image_3.tif]

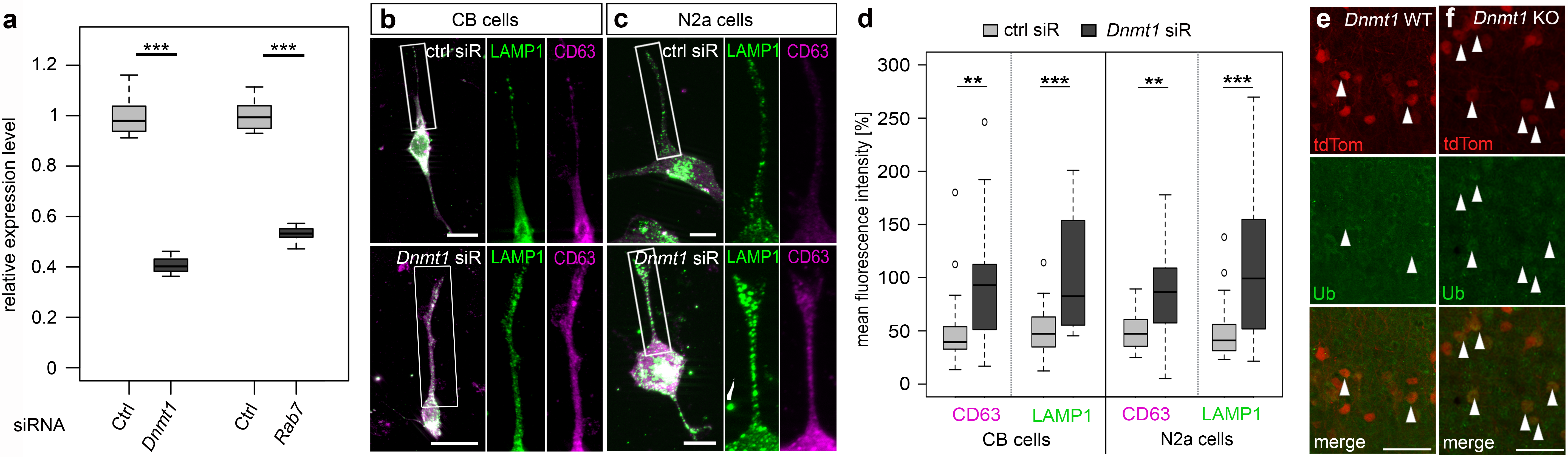

Supplement: Supplementary file 5 [file Image_4.tif]
